# Therapeutic potential of inhibiting histone 3 lysine 27 demethylases: a review of the literature

**DOI:** 10.1186/s13148-022-01305-8

**Published:** 2022-08-01

**Authors:** Jeries Abu-Hanna, Jigisha A. Patel, Evangelos Anastasakis, Richard Cohen, Lucie H. Clapp, Marilena Loizidou, Mohammad M. R. Eddama

**Affiliations:** 1 Division of Surgery and Interventional Science, Research Department of Surgical Biotechnology, University College London, GI Services, Ground Floor, 250 Euston Road, London, NW1 2PG UK; 2 UCL Medical School, University College London, London, UK; 3 Department of Gastroenterology, University College London Hospital, London, UK; 4 Institute of Cardiovascular Science, University College London, London, UK

**Keywords:** Epigenetics, H3K27, Histone lysine demethylase, UTX, JMJD3, KDM6A, KDM6B, Cancer, Inflammation, Autoimmune diseases, Infectious diseases, GSK-J4

## Abstract

Histone 3 lysine 27 (H3K27) demethylation constitutes an important epigenetic mechanism of gene activation. It is mediated by the Jumonji C domain-containing lysine demethylases KDM6A and KDM6B, both of which have been implicated in a wide myriad of diseases, including blood and solid tumours, autoimmune and inflammatory disorders, and infectious diseases. Here, we review and summarise the pre-clinical evidence, both in vitro and in vivo, in support of the therapeutic potential of inhibiting H3K27-targeting demethylases, with a focus on the small-molecule inhibitor GSK-J4. In malignancies, KDM6A/B inhibition possesses the ability to inhibit proliferation, induce apoptosis, promote differentiation, and heighten sensitivity to currently employed chemotherapeutics. KDM6A/B inhibition also comprises a potent anti-inflammatory approach in inflammatory and autoimmune disorders associated with inappropriately exuberant inflammatory and autoimmune responses, restoring immunological homeostasis to inflamed tissues. With respect to infectious diseases, KDM6A/B inhibition can suppress the growth of infectious pathogens and attenuate the immunopathology precipitated by these pathogens. The pre-clinical in vitro and in vivo data, summarised in this review, suggest that inhibiting H3K27 demethylases holds immense therapeutic potential in many diseases.

## Introduction

Epigenetics refers to heritable changes in gene expression that are not a consequence of changes in the DNA sequence [1]. By precisely regulating gene expression, epigenetics can determine the phenotype and fate of a given cell and, if dysregulated, can lead to inappropriate gene expression, giving rise to pathological cell behaviour [2]. In the nuclei of eukaryotic cells, about two turns of DNA are wrapped around a histone octamer (typically composed of two copies of each of H2A, H2B, H3 and H4) to form a nucleosome, the basic structural unit of chromatin [3, 4]. This structural arrangement enables chromatin to be organised into either condensed, transcriptionally silent regions called heterochromatin or loose, transcriptionally active regions called euchromatin [5]. Epigenetic processes serve to remodel the structure of chromatin to regulate the accessibility of genes to transcriptional factors and involve DNA methylation and post-translational covalent modifications of histones, namely phosphorylation, ubiquitination, acetylation, SUMOylation, crotonylation and methylation [3]. These epigenetic events can be influenced by environmental cues or signals, allowing interactions between the environment and the genome [6].

Methylation of histones on lysine residues plays a pivotal role in regulating gene expression. Lysine residues within the protruding N-terminal tails of histones can be monomethylated (me1), dimethylated (me2) or trimethylated (me3) [7]. To date, the most extensively investigated methylation sites include histone 3 lysine 4 (H3K4), H3K9, H3K27, H3K36, H3K79 and H4K20 [3, 7]. Generally, methylation of H3K4, H3K36 and H3K79 induces an open chromatin structure to drive gene expression [8]. Methylation of H3K9, H3K27 and H4K20, on the other hand, is frequently associated with a compact chromatin conformation and suppression of gene expression [7]. Methylation of lysine residues, which is mediated by lysine methyltransferases (KMTs), was initially considered to be a stable post-translational event [7, 9]. However, the discovery of the H3K4 lysine demethylase 1A (KDM1A; also known as LSD1) unveiled the reversibility of histone methylation [10]. To methylate histones, KMTs catalyse the transfer of methyl groups from S-adenosylmethionine (SAM) to the amino groups of lysine residues within the exposed *N*-terminal tails [3]. Histone demethylation is mediated by lysine demethylases (KDMs), which can be categorised into two families: amino oxidase homologue lysine demethylase 1 (KDM1) and Jumonji C (JmjC) domain-containing KDMs [11].

**Fig. 1:**
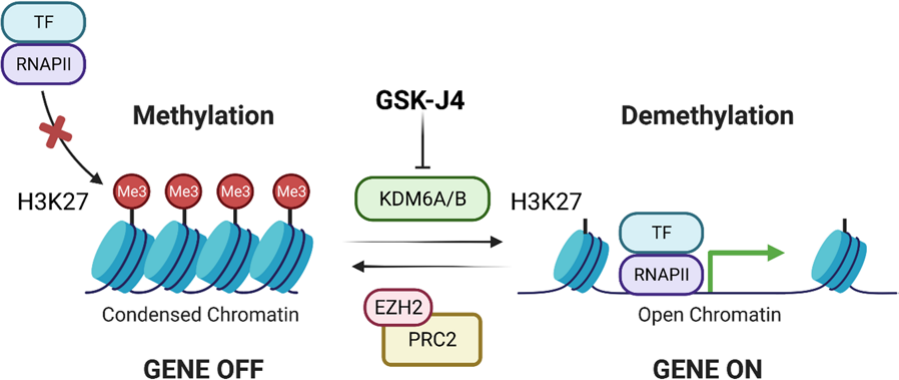
Trimethyl H3K27 constitutes a repressive epigenetic mark of gene expression. When trimethylated at lysine 27 (K27) within its N terminus, histone 3 (H3) is intimately associated with inactive gene promoter regions, where the binding of RNA polymerase II (RNAPII) and transcription factors (TFs) is hampered. Trimethylation of H3K27 is unique in that it is exclusively catalysed by the H3K27-specific methyltransferase, enhancer of zeste homologue 2 (EZH2), a catalytic component of the polycomb repressive complex 2 (PRC2). Demethylation of H3K27, namely by the H3K27-specific lysine demethylases 6A (KDM6A) and 6B (KDM6B), serves to activate gene transcription by permitting the binding of RNAPII and TFs to promoter regions. This figure was created in BioRender.com

Trimethylation of H3K27, particularly at gene promoter regions and transcriptional start sites, serves as an important epigenetic mark of gene silencing and, compared to the similarly gene repressive H3K9 methylation, is easily reversible (Fig. 1) [12]. H3K27 demethylation and subsequent trimethylation are exclusively mediated by the KMT, enhancer of zeste homologue 2 (EZH2), which is an enzymatic component of the polycomb repressive complex 2 (PRC2). Trimethyl H3K27 (H3K27me3) promotes the recruitment and stabilisation of PRC1, which silences gene expression in several ways, including compaction of chromatin to reduce accessibility of the promoter region to transcription factors and monoubiquitylation of H2AK119 [13, 14]. Through its EED domain, PRC1 is also able to complex with DNA methyltransferases and histone deacetylases, further consolidating the transcriptionally silent state [3]. The H3K27me3 epigenetic mark is self-maintaining such that, during DNA replication, H3K27me3 recruits PRC2 and PRC1 complexes to the nucleosomes of nascent DNA strands to sustain gene silencing [15, 16]. Demethylation of H3K27 is catalysed by two members of the KDM6 family of lysine demethylases: JmjC domain-containing 3 (JMJD3; also known as KDM6B) and ubiquitously transcribed tetratricopeptide repeat on X chromosome (UTX; also known as KDM6A) [3].

In disease, the amount and distribution of H3K27me3 can be altered due to the abnormal expression and/or activity of either the methyltransferase EZH2 or the demethylases KDM6A and KDM6B. This review specifically addresses the role of H3K27 demethylation in various diseases, including cancer, inflammatory diseases and viral and bacterial infections, and the therapeutic potential of inhibiting this demethylation, focusing on the small-molecule KDM6A/B inhibitor GSK-J4. Table 1 summarises the pre-clinical in vitro and in vivo evidence in support of the therapeutic potential of GSK-J4.

**Table 1:** In vitro and in vivo pre-clinical evidence in support of the therapeutic potential of GSK-J4 in various diseases

Disease	In vitro evidence	In vivo evidence	References
*Malignancies*			
Acute myeloid leukaemia (AML)	GSK-J4 reduces proliferation and colony-forming ability of primary AML cells and AML cell lines	GSK-J4 displays anti-tumour activity in AML xenograft mouse model	[24]
	GSK-J4 dose-dependently induces cell cycle arrest in the S phase in AML cell lines		[92]
T cell acute lymphoblastic leukaemia (T-ALL)	GSK-J4 suppresses the growth of T-ALL cell lines and primary T-ALL cells by inducing cell cycle arrest and apoptosis	–	[28]
Colorectal cancer	GSK-J4 sensitises colorectal cancer cells to oxaliplatin-induced apoptosis	GSK-J4 reduces tumour volume in mice injected with oxaliplatin-resistant patient-derived xenografts	[34]
	GSK-J4 sensitises colorectal cancer cells to fluorouracil		
Prostate cancer	GSK-J4 inhibits proliferation of castration-resistant prostate cancer cell lines	–	[37]
	GSK-J4 sensitises castration-resistant prostate cancer cell lines to cabazitaxel		
Breast cancer	GSK-J4 inhibits the self-renewal capacity and colony-forming ability of breast cancer stem cells	GSK-J4 suppresses tumour growth in breast cancer xenograft mouse model	[39]
	GSK-J4 sensitises luminal breast cancer cell lines to phosphoinositide 3-kinase inhibitors		[41]
Neuroblastoma	GSK-J4 reduces viability in neuroblastoma cell lines	GSK-J4 blocks growth of chemorefractory and patient-derived xenografts in mice	[31]
Gliomas	GSK-J4 inhibits proliferation of native and temozolomide-resistant glioblastoma cells by blocking cell cycle progression into G2 phase	GSK-J4 improves survival in mice with H3K27 mutation-harbouring diffuse intrinsic pontine glioma xenografts	[50]
*Inflammatory conditions*			
Rheumatoid arthritis (RA)	GSK-J4 inhibits PDGFBB-induced proliferation and migration of fibroblast-liked cnidocytes	GSK-J4 ameliorates joint swelling and bone erosion and destruction in mouse model of collagen-induced arthritis	[60]
	GSK-J4 inhibits IFNγ production in natural killer cells derived from peripheral bloods and synovial fluids of patients with RA		[53]
	GSK-J4 inhibits the ability of NK cells to promote the formation of osteoclasts, which mediate bone erosion in RA		[53]
	GSK-J4 inhibits TNFα production in macrophages from patients with RA		[18]
Osteoarthritis	GSK-J4 disrupts collagen (COL2A1 and COL10A1) and glycosaminoglycan (aggrecan) synthesis in chondrogenic mesenchymal stem cells	GSK-J4 protects against cartilage erosion in destabilisation of medial meniscus (DMM) murine model of osteoarthritis	[63]
	GSK-J4 inhibits the expression of cartilage destroying proteases MMP9, MMP13 and ADAMTS5 in human articular chondrocytes		[63]
Inflammatory bowel disease	GSK-J4 promotes tolerogenic capacity of dendritic cells in vitro	GSK-J4 ameliorates severity of dextran sodium sulphate (DSS)-induced colitis in mice	[68]
		Dendritic cell treated ex vivo with GSK-J4 attenuates DSS-induced colitis in mice by promoting CD4^+^ T cell polarisation towards Treg rather than Th17	
Atherosclerosis	GSK-J4 inhibits PDGFBB-induced proliferation and migration of aortic smooth muscle cells	GSK-J4 attenuates neointimal formation in mice with carotid artery ligation	[71]
Multiple sclerosis	–	GSK-J4 reduces severity of experimental autoimmune encephalomyelitis in mice	[56]
*Pathogens*			
Schistosomiasis	GSK-J4 dose-dependently reduces motility and viability of adult schistosomal worms and drug-resistant schistosomula	–	[91]
Respiratory syncytial virus (RSV)	GSK-J4 inhibits antigen-presenting capacity of bone marrow-derived and pulmonary dendritic cells infected with RSV	GSK-J4 ameliorates pulmonary immunopathology associated with RSV infection in mice	[77]
Herpes simplex virus 1 (HSV1)	GSK-J4 inhibits induced reactivation of HSV1 from latency in primary adult murine trigeminal ganglion neurons	–	[85]
Human immunodeficiency virus 1 (HIV1)	GSK-J4 inhibits induced reactivation of HIV1 from latency in T cells	–	[82]
*Escherichia coli* (*E. coli*)	GSK-J4 inhibits LPS-induced expression of pro-inflammatory cytokines in macrophages derived from the peritoneum of septic mice infected with E. coli	GSK-J4 protects against early sepsis and improves survival in mice injected with E. coli strains from human clinical specimens	[88]

## Discovery and mechanism of action of GSK-J4

Kruideneir et al. [17] discovered GSK-J1, the first selective and potent histone demethylase inhibitor, by screening a library of GlaxoSmithKline corporate compounds (~ 2 million) for their ability to inhibit KDM6B, a member of the KDM6 subfamily of H3K27 demethylases, and optimising a series of weakly active hits. GSK-J1 inhibited KDM6B with a half-maximum inhibitory concentration (IC_50_) of 60 nM [17]. It was also found to inhibit KDM6A but was inactive against a panel of other demethylases of the JMJ family, protein kinases and unrelated proteins [17]. GSK-J1 was later shown to exhibit 5–10-fold lower inhibitory activity against KDM5 demethylases, particularly KDM5B and KDM5C, suggesting that GSK-J1 cannot be solely used to draw conclusions with respect to the role played by H3K27-specific demethylases in biological processes or disease pathogeneses and that there remains a need for more selective inhibitors [18]. Co-crystallisation studies of GSK-J1 bound to KDM6B revealed that GSK-J1 is a competitive inhibitor of α-ketoglutarate and Fe^2+^, two cofactors required for KDM6B enzymatic activity [17]. With similar physicochemical properties to GSK-J1 and no H3K27 demethylase inhibitory activity, the regio-isomer GSK-J2 was developed to serve as an inactive control molecule. Both GSK-J1 and GSK-J2 possess a highly polar carboxylate group that restricts their ability to permeate cell membranes and achieve pharmacologically relevant intracellular concentrations [17]. A pro-drug approach was therefore employed to mask the polarity of this acid group with an ethyl ester and render GSK-J1 and GSK-J2 lipophilic [17]. This yielded the pro-drugs GSK-J4 and GSK-J5, allowing membrane permeation and subsequent hydrolysis to GSK-J1 and GSK-J2, respectively, by cytoplasmic esterases [17].

## H3K27 demethylase inhibition in malignancies

Cancer is a complex disease, characterised by uncontrolled proliferation, apoptosis resistance and poor cellular differentiation [19]. The malignant transformation of cells in cancer involves the transcriptional activation of oncogenic genes and/or the transcriptional suppression of tumour suppressor genes [20]. Overwhelming evidence incriminates KDM6A/B-mediated H3K27 demethylation in the transcriptional dysregulation of oncogenes and tumour suppressor genes that underlies this pathological cellular transformation. This section provides an overview of the role of KDM6A/B in the pathogenesis of various solid and haematological malignancies and pre-clinical evidence supporting the use of GSK-J4 as an anticancer therapeutic.

### Acute myeloid leukaemia

**Fig. 2:**
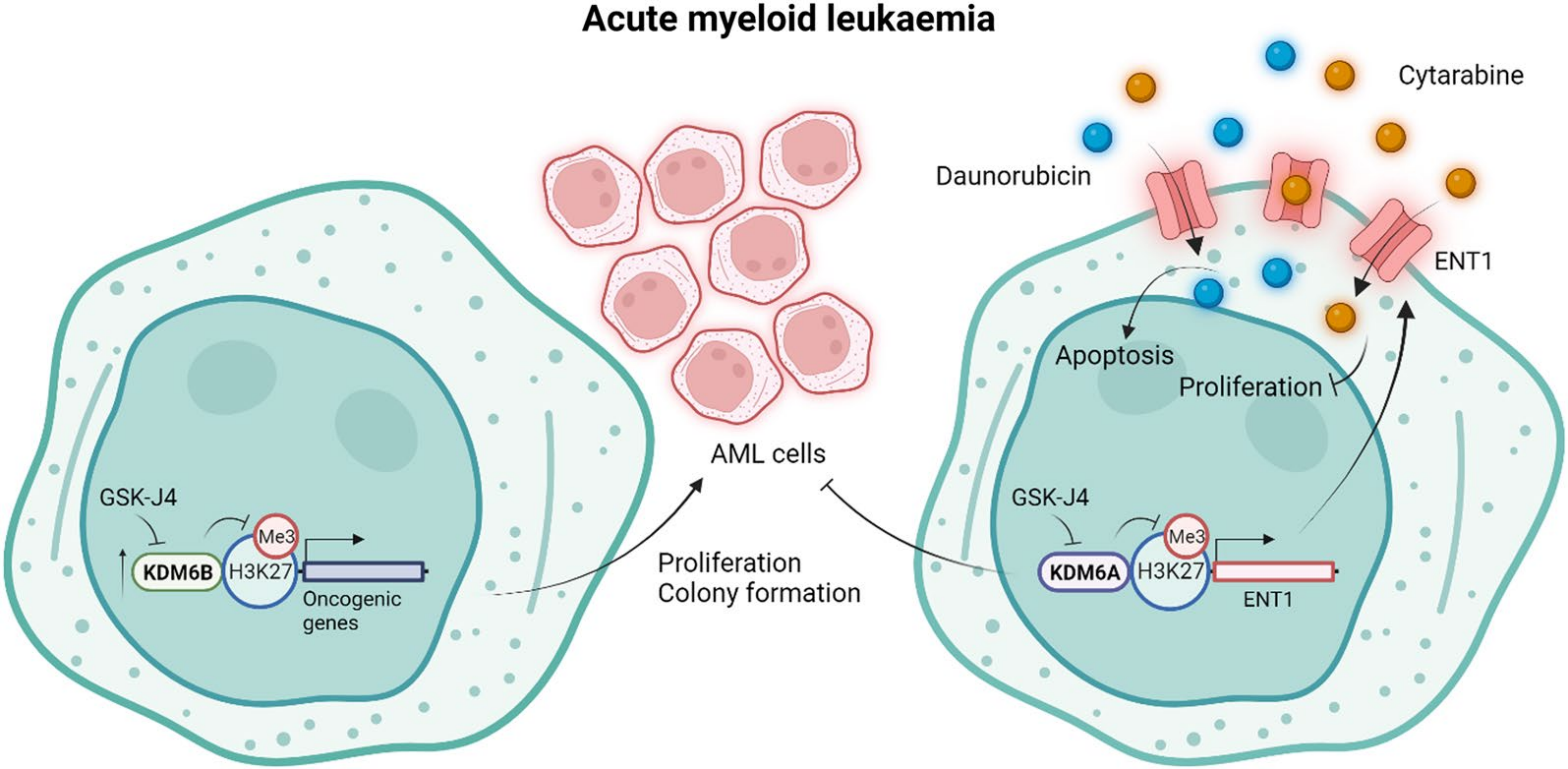
KDM6A/B inhibition in acute myeloid leukaemia. KDM6A and KDM6B have contrasting roles in the pathogenesis of acute myeloid leukaemia (AML). Increased KDM6B expression and occupancy at gene promoter regions in AML drives the transcription of genes involved in regulating the cell cycle, DNA replication, apoptosis and cellular differentiation, leading to the proliferation and colony formation of AML cells. Inhibition of KDM6B therefore attenuates AML cell proliferation and colony formation by promoting repressive H3K27 trimethylation, which downregulates the expression of these oncogenic genes. Contrastingly, through H3K27 demethylation, KDM6A upregulates the expression of the drug influx transporter ENT1, allowing the cellular entry of the chemotherapeutic agents cytarabine and daunorubicin and the consequent inhibition of AML cell proliferation and colony formation. Inhibition of KDM6A with GSK-J4 renders AML cells resistant to these chemotherapeutic agents by suppressing their cellular uptake through the downregulation of ENT1. This figure was created in BioRender.com

Acute myeloid leukaemia (AML) is a heterogeneous neoplastic disorder characterised by the clonal expansion of myeloid precursor cells and accounts for 80% of acute leukaemia cases [21]. Complete remission is achievable with classical chemotherapy in most AML patients; however, the relapse rate remains high (~ 40% of AML patients) and five-year survival rates (30–35% in AML patients up to 60 years and 10–15% in older patients) poor [22, 23]. There remains a need for novel targets to improve patient outcomes. Overexpression of KDM6B was observed in multiple AML cell lines as well as in primary bone marrow-derived mononuclear cells from patients with AML [24]. High KDM6B expression was also associated with poor overall survival in AML patients [24]. Inhibition of KDM6B with GSK-J4 attenuated the proliferation and colony formation of both primary AML cells and AML cell lines by increasing global levels of the repressive mark H3K27me3 (Fig. 2) [24]. Bulk RNA sequencing (RNA-seq) and pathway enrichment analyses revealed an effect of GSK-J4 on genes involved in regulating the cell cycle, DNA replication, cell differentiation and apoptosis [24]. Treatment with GSK-J4 also attenuated disease development in a mouse model with a human AML xenograft [24].

In contrast to KDM6B, which seems to activate oncogenic gene expression to support AML pathogenesis, KDM6A has recently been shown to play a tumour suppressor role in AML [25]. Stief et al. (2020) [25] reported inactivating mutations in KDM6A in patients with relapsed AML. They also demonstrated that AML cells devoid of KDM6A were more resistant to treatment with the chemotherapeutic agents cytarabine and daunorubicin, a consequence of reduced expression of the drug influx transporter ENT1, and that re-expression of KDM6A supressed proliferation and restored responses to cytarabine (Fig. 2) [25]. These findings indicate that KDM6A confers upon AML cells sensitivity to chemotherapeutic agents and that inhibition of KDM6A with GSK-J4 in AML patients on chemotherapy might facilitate the emergence of resistant clones during relapse [25].

### T cell acute lymphoblastic leukaemia

**Fig. 3:**
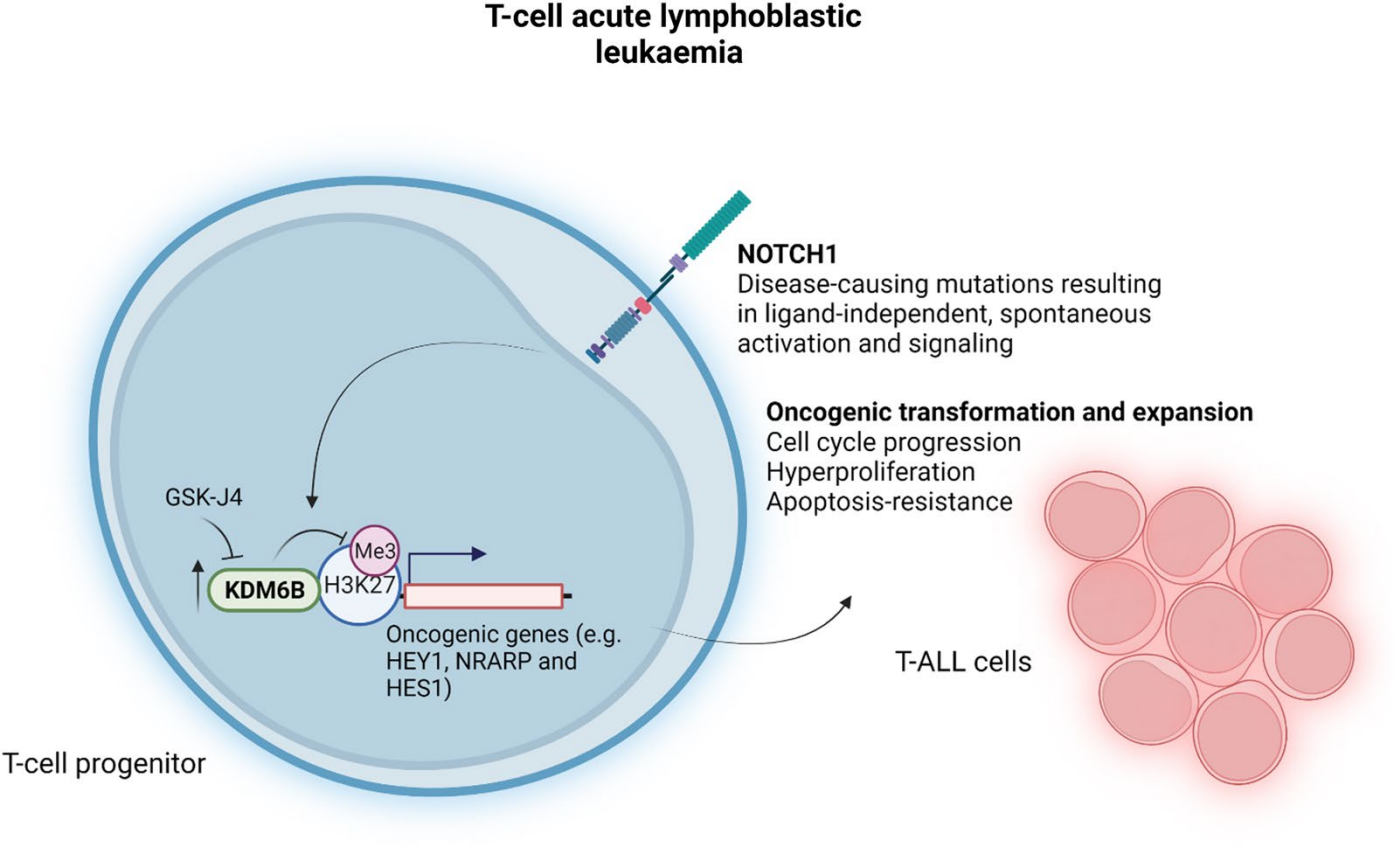
KDM6B inhibition in T cell acute lymphoblastic leukaemia. T cell acute lymphoblastic leukaemia (T-ALL) involves the oncogenic transformation and expansion of T cell progenitors. T-ALL cells overexpress KDM6B, which predominantly occupies the promoter regions of oncogenic genes that are targets of NOTCH1 signalling, including HEY1, NRARP and HES1. Indeed, in T-ALL, mutations in the NOTCH1 receptor result in ligand-independent, spontaneous receptor activation and signalling. GSK-J4 suppresses the growth of T-ALL cells by increasing the levels of the repressive mark H3K27me3 without influencing KDM6B occupancy. This figure was created in BioRender.com

T cell acute lymphoblastic leukaemia (T-ALL) is a haematological malignancy involving the oncogenic transformation and expansion of T cell progenitors [26]. T-ALL has a poor overall prognosis and a high relapse rate (~ 20% of T-ALL patients) due to the availability of very few non-cytotoxic targeted therapies [27]. Compared to normal T cell progenitors, T-ALL cells were found to overexpress KDM6B, implicating KDM6B in the oncogenesis of T-ALL (Fig. 3) [28]. Contrastingly, KDM6A remained unaltered in T-ALL cells and was found to play a tumour suppressor role as suggested by the identification of somatic loss-of-function mutations in paediatric and adult T-ALL patients as well as the finding that KDM6A deficiency hastened T-ALL progression in a NOTCH1-overexpressing murine model of T-ALL [28, 29]. Chromatin immunoprecipitation followed by sequencing revealed that, in the human T-ALL cell line CUTTL1, KDM6B was primarily bound to NOTCH1 target genes, including the oncogenic HEY1, NRARP and HES1 genes [23]. Accordingly, inhibition of KDM6B with GSK-J4 suppressed the growth of T-ALL cell lines, as well as primary T-ALL cells, by inducing cell cycle arrest and apoptosis [28]. This growth-suppressive effect was coupled with increased H3K27me3 levels at repressed genes but no change in KDM6B occupancy, underpinning the ability of GSK-J4 to inhibit the demethylase activity of KDM6B rather than its recruitment to gene promoter regions [28].

### Neuroblastoma

Despite advances in therapy, high-risk neuroblastoma remains a fatal cancer that arises as a result of the failure of neural crest cells to differentiate [30]. High-throughput drug screening demonstrated potent anti-tumour activity of GSK-J4 in neuroblastoma cell lines among other solid cancer cell lines [30]. Bulk RNA-seq revealed an effect of GSK-J4 on pathways involved in neuronal differentiation and neuritogenesis in neuroblastoma cells, particularity those deemed sensitive to GSK-J4 [30]. For example, treatment with GSK-J4 upregulated the critical differentiation genes ENO2, CHD5, NGF and NRG1, while downregulating the expression of inhibitors of differentiation, including ASCL1 and MYCN [30]. In addition to its positive effect on differentiation, GSK-J4 induced apoptosis and/or necrosis of neuroblastoma cells as evidenced by annexin V/propidium iodide staining and increased caspase-3 activity [30]. Bulk RNA-seq showed upregulation of p53 upregulated modulator of apoptosis (PUMA) in GSK-J4-treated neuroblastoma cells [30]. This increase in PUMA expression was shown to be independent of p53 stabilisation and alternatively a consequence of endoplasmic reticulum stress [30].

An independent study by Yang et al. (2019) provided evidence in support of an anti-tumorigenic function of KDM6B in neuroblastoma, contradicting the findings of Lochmann et al. (2018) [30, 31]. Yang et al. (2019) demonstrated an association between low KDM6B and poor prognosis in patients with high-risk neuroblastoma [31]. They also showed that ectopic overexpression of KDM6B considerably inhibited the proliferation of human neuroblastoma cell lines and promoted their neuronal differentiation as implied by extensive outgrowth of neurites, including neuronal axons and dendrites, associated with the upregulation of the neuronal differentiation-promoting genes NEFM, GFRA3 and RET [31].

### Colorectal cancer

**Fig. 4:**
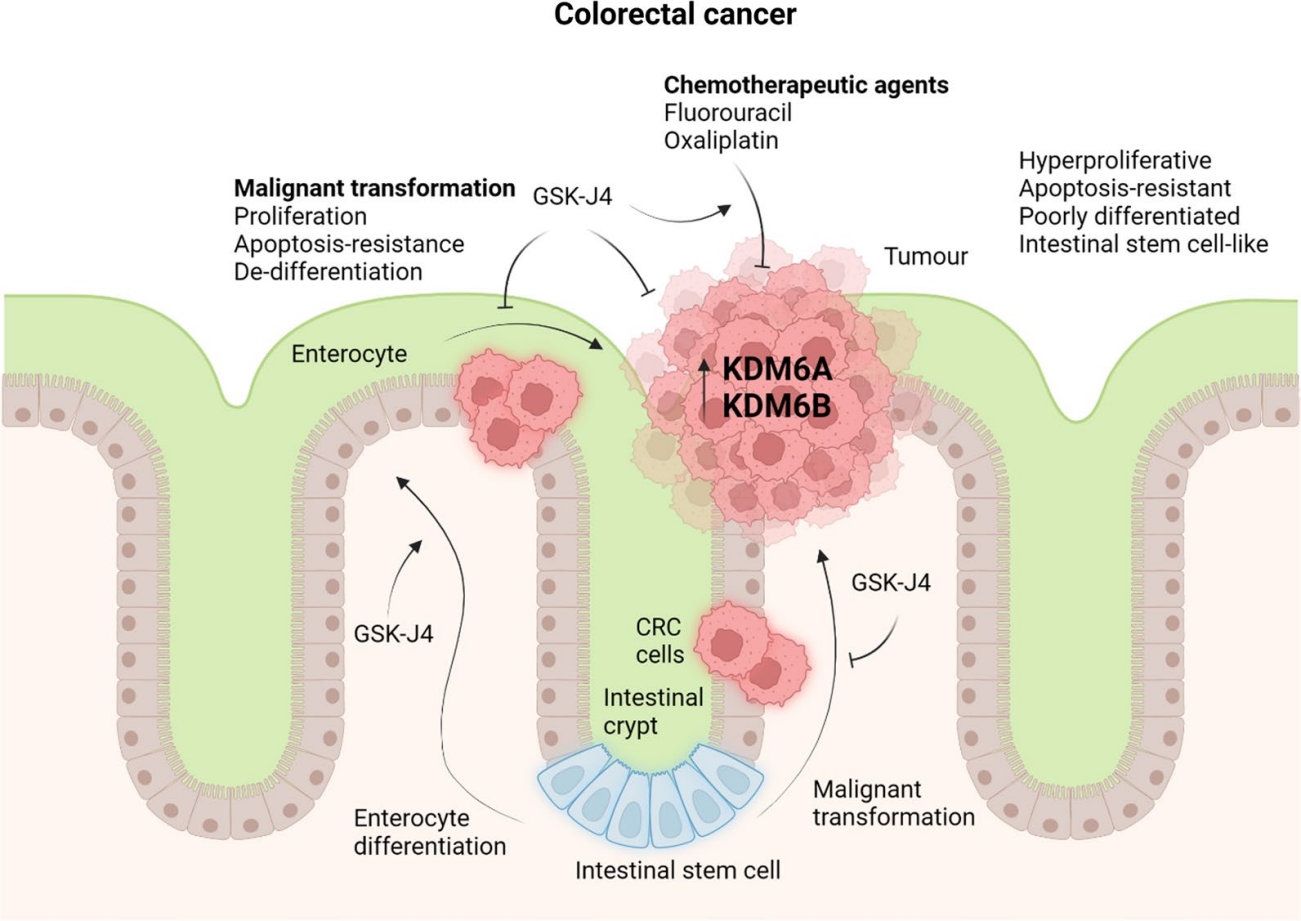
KDM6A/KDM6B inhibition in colorectal cancer. KDM6A/B expression negatively correlates with survival in patients with colorectal cancer (CRC), suggesting a pathogenic role for these H3K27 demethylases. Inhibition of KDM6A/B with GSK-J4 attenuates the hyperproliferative, apoptosis-resistant and stem cell-like phenotype of CRC cells and promotes their enterocyte differentiation. GSK-J4 also inhibits the malignant transformation of intestinal stem cells, which reside in the intestinal crypts and are thought to be the source of most CRC cells, promoting instead their differentiation into enterocytes. In addition to its direct tumoricidal activity, GSK-J4 heightens the sensitivity of colorectal cancer cells to the chemotherapeutic agents fluorouracil and oxaliplatin. This figure was created in BioRender.com

Colorectal cancer (CRC) remains a highly morbid and fatal cancer with no completely curative therapy and a 5-year survival rate of only 10% [32, 33]. CRC is the third most prevalent cancer worldwide, accounting for 10% of all cancers and cancer-related deaths worldwide [32]. KDM6A/B expression has been shown to negatively correlate with survival in patients with CRC, supporting the rationale that inhibiting KDM6A/B may be beneficial for patients with CRC (Fig. 4) [33]. Indeed, GSK-J4 suppressed the proliferation of CRC cells obtained from various sites along the bowel. Interestingly, CRC cells derived from the large intestine, the predominant site of CRC pathology, exhibited the greatest sensitivity to GSK-J4 compared to CRC cells from other sites [33]. GSK-J4 also inhibited the migratory capacity of CRC cells and limited the growth of subcutaneous patient-derived xenografts in mice [33]. GSK-J4 sensitised CRC cells to the chemotherapeutic agent 5-fluorouracil, and the synergy between GSK-J4 and 5-fluorouracil suggests that GSK-J4 may also enhance the clinical efficacies of existing chemotherapeutic agents [33]. A separate study by Wang et al. (2020) demonstrated that KDM6A/B silencing or inhibition with GSK-J4 sensitised CRC cells to another chemotherapeutic agent oxaliplatin and co-administration of GSK-J4 and oxaliplatin suppressed tumour growth in an oxaliplatin-resistant patient-derived CRC xenograft murine model [34]. Conversely, reducing H3K27 trimethylation via the inhibition of EZH2 rendered CRC cells resistant to oxaliplatin [34], further supporting the notion that elevating the levels of the H3K27me3 could sensitise chemotherapy-resistant CRC cells.

Intestinal stem cells (ISCs) residing in the crypts of the intestinal villi maintain the high turnover of the intestinal epithelium and are suspected to be the source of most, if not all, CRCs due to their proliferative and self-restorative behaviour [33]. Indeed, CRC cells have been shown to exhibit a phenotype akin to that of ISCs [33]. In murine intestines, the expression of KDM6A and KDM6B, the targets of GSK-J4, was higher in the crypts, where ISCs reside, than in the villi, which are lined by differentiated intestinal epithelial cells or enterocytes [33]. Transcriptomic and pathway enrichment analyses revealed that GSK-J4 downregulates the expression of genes, which regulate the cell cycle, DNA repair and Wnt signalling pathway, while upregulating the expression of genes associated with differentiated enterocytes and the MAPK, TNF, p53 and apoptosis pathways [33]. These findings suggest that GSK-J4 through inhibiting KDM6A and KDM6B possesses the ability to suppress the proliferative and ISC-like phenotype of CRC cells, promoting enterocyte differentiation.

### Prostate cancer

Prostate cancer is the second most common malignancy in males worldwide, the growth of which is predominantly driven by steroid androgens [35]. Consequently, locally advanced and metastatic prostate cancers are treated with androgen ablation therapy that serves to inhibit the production of endogenous testosterone or block the activation of androgen receptors [35]. Despite the initial effectiveness of these hormone therapies, they offer only a transient relief with a high likelihood of lethal recurrence in the form of castration-resistant prostate cancer [36]. Overexpression of KDM6A/B and a reduction in the global levels of H3K27me2/3 were reported in prostate cancer and strongly correlate with disease aggressiveness [37]. Inhibition of KDM6A/B with GSK-J4 has been shown to inhibit the proliferation of prostate cancer cell lines with castration-resistant prostate cancer cell lines, specifically those lacking the androgen receptor, exhibiting the highest sensitivity [37]. These anti-proliferative effects of GSK-J4 were accompanied by a reduction in the global levels of H3K27me1 and an increase in those of H3K27me3 [37]. Moreover, GSK-J4 sensitised castration-resistant prostate cancer cells to cabazitaxel, one of few therapeutics approved for treatment of castration-resistant prostate cancer [37].

### Breast cancer

Breast cancer is the most common malignancy in females with approximately one new case diagnosed every 18 s [38]. Advances in multimodal therapy and early detection improved chances for cure in 70–80% of patients with early breast cancer [38]. However, relapse and subsequent death occur in a significant proportion (~ 30%) of these patients [39, 40]. Relapse has been proposed to occur as a consequence of the existing breast cancer stem cells (BCSCs), which possess limitless proliferative potential and are particularly resistant to clinically employed therapy [39]. There remains an unmet need for targeting BCSCs to prevent this potentially fatal relapse. Inhibiting KDM6A/B with GSK-J4 has been shown to inhibit the proliferation of the luminal breast cancer cell lines MCF7 and MDA-MB-231, with the poorly differentiated triple-negative MDA-MB-231 cells showing greater resistance to the anti-proliferative effect of GSK-J4 [39]. Moreover, GSK-J4 attenuated the self-renewal capacity and colony-forming ability of BCSCs present in mammosphere cultures (non-adherent, serum-free) of MCF7 and MDA-MB-231 cells [39]. Interestingly, these mammosphere BCSCs expressed higher transcript levels of KDM6A and KDM6B than adherent BC cells [39]. Finally, concordant with its anti-tumourigenic effects in vitro, GSK-J4 suppressed tumour growth in mice whose mammary fat pads were inoculated with MDA-MB-231 cells [39]. These data suggest that GSK-J4 could be used to eradicate residual BCSCs and prevent relapse in patients with breast cancer.

Inhibition of KDM6B with GSK-J4 has been demonstrated to sensitise luminal breast cancer cell lines, including those that harbour mutations in the PIK3CA gene, to apoptosis induced by phosphoinositide 3-kinase (PI3K) inhibitors, which have shown limited or suboptimal efficacy in late-phase clinical trials in luminal breast cancers with PIK3CA mutations [41]. The mechanism by which GSK-J4 sensitises breast cancer cells to PI3K inhibitor involves silencing the IGFBP5 gene, which confers a growth advantage upon PI3K inhibitor-resistant breast cancer cells [41]. GSK-J4 could, therefore, be used as an adjunct therapy to enhance the clinical efficacy of PI3K inhibitors in breast cancer patients. In addition to its apparent oncogenicity, KDM6B has been demonstrated to act as a tumour suppressor, suggesting a context-dependent role for KDM6B in the pathogenesis of breast cancer [42]. Xun et al. (2021) recently showed that the prognosis of breast cancer patients with low KDM6B expression was worse than that of patients with high KDM6B expression. KDM6B overexpression was also shown to inhibit the proliferation, colony-forming ability and migratory capacity of breast cancer cells in vitro [42]. Inoculation of breast fat pads in mice with KDM6B-overexpressing MDA-MB-231 cells resulted in smaller tumours and fewer metastatic lung nodules, supporting a tumour suppressor role for KDM6B in the growth and metastasis of breast cancer in vivo [42].

### Gliomas

Gliomas constitute a heterogeneous group of primary brain tumours and account for 30% of all brain tumours and 80% of malignant ones [43]. Glioblastoma is a rare type of glioma that accounts for 4% of all tumour-related deaths, making it one of the deadliest human tumours [44]. Glioblastomas and other gliomas are thought to originate from neuroglial progenitor cells [45]. Despite advances in multimodal therapy, incorporating surgery, radiation therapy and chemotherapeutic agents, such as temozolomide, prognosis in patients with gliomas remains poor, and the chances of long-term survival are slim [46]. Interestingly, some paediatric gliomas, including diffuse intrinsic pontine gliomas (DIPGs), are associated with somatic mutations in histone 3 variant H3.3 at K27, which lead to global H3K27 hypomethylation through the sequestration and inactivation of PRC2 and its KMT component EZH2 [47]. KDM6A/B inhibition with GSK-J4 rescued the hypomethylating effect of H3K27 mutations and improved survival in mice with H3K27 mutation-harbouring DIPG xenografts [48]. Increased expression of KDM6B and consequent global H3K27 demethylation have also been reported in glioblastoma tissues and glioblastoma cell lines, implicating the H3K27 demethylation in the pathogenesis of this neoplastic disorder [49]. GSK-J4 restored H3K27 trimethylation and exerted anti-proliferative, pro-apoptotic and anti-migratory effects on the glioblastoma cell lines U87 and U251 [50]. GSK-J4 has also been shown to sensitise glioblastoma cell lines to the chemotherapeutic agent temozolomide, suggesting that GSK-J4 could be used to enhance the efficacy of current therapeutics [50].

## H3K27 demethylase inhibition in inflammatory and autoimmune conditions

Mounting evidence suggests that epigenetic mechanisms, including H3K27 demethylation by the demethylases KDM6A and KDM6B, are involved in mediating inflammatory responses by regulating the functions of various immune effector cells of both the innate and adaptive immune systems. In this section, we review the role of KDM6A and KDM6B in regulating the activity of key cellular players in inflammation and the pre-clinical evidence that supports the potential use of the KDM6A/B small-molecule inhibitor GSK-J4 in alleviating inflammatory and autoimmune conditions.

### Inflammatory cells

Macrophage activation leads to the production of a myriad of pro- and anti-inflammatory cytokines, including interleukin-1β (IL-1β), tumour necrosis factor α (TNFα) and IL-10 [51]. Several lines of evidence implicate H3K27 demethylases, namely KDM6B, in the inflammatory activation of macrophages and the consequent production of cytokines. In human primary macrophages, KDM6B expression is rapidly induced secondary to nuclear factor-κB (NF-κB) activation by pro-inflammatory stimuli, namely lipopolysaccharide (LPS), and recruited to transcription start sites of over 70% of LPS-responsive genes, promoting macrophage polarisation towards the pro-inflammatory M1 phenotype [17]. It is important to note here that, despite not being induced by LPS, KDM6A can compensate for the absence of KDM6B in the macrophage response to LPS as evidenced by unaltered LPS responses in human and murine macrophages devoid of KDM6B [17, 52]. Consistently, GSK-J4 suppressed LPS-induced TNFα production in macrophages by promoting the accumulation of H3K27me3 on the transcription start site of the TNFA gene [17]. This effect of GSK-J4 is not restricted to TNFA as other LPS-responsive genes have been shown to respond to the GSK-J4-mediated inhibition of H3K27 demethylation [17].

KDM6B has also been shown to be critical in driving the functional polarisation of murine macrophages to M2 cells in response to chitin and helminthic infection via regulating the expression of IRF4, a key transcriptional factor for M2 macrophage polarisation [52]. M2 macrophages are important players in the host defence against parasitic, helminthic and fungal infections and inhibiting KDM6B may therefore render the host susceptible to these pathogens. Interestingly, KDM6B was found to be dispensable for the M1 polarisation of murine macrophages in response to bacterial infection and pro-inflammatory stimulation [52]. This is likely due to the ability of KDM6A to compensate for the absence of KDM6B as described by Kruidenier et al. (2012) in human macrophages [17].

The primary role of natural killer (NK) cells is to destroy virally infected or malignantly transformed cells by inducing their lysis [53]. However, it is now recognised that, in addition to their lytic effector function, NK cells partake in inflammatory processes, such as those observed in autoimmune diseases, by releasing cytokines and growth factors [53]. Like in macrophages, H3K27 demethylation by KDM6A and KDM6B in NK cells induces an inflammatory phenotype, characterised by increased production of TNFα and interferon-γ (IFNγ) [53]. Inhibition of KDM6A/B with GSK-J4 or RNA interference reduces the expression and subsequent production of TNFα and IFNγ following stimulation with IL-15 by increasing the level of repressive H3K27me3 enrichment across the transcription start sites of both the TNFA and IFNG genes [53]. Moreover, RNA-seq revealed a strong effect of GSK-J4 inhibition of KDM6A/B on inflammatory genes other than those encoding TNFα and IFNγ in IL-15-stimulated NK cells as well as genes involved in various cellular processes, including the cell cycle and metabolism [53]. In addition to their intrinsic ability to generate TNFα, NK cells can induce the production of TNFα in macrophages through direct cellular contact [53]. This is supported by the finding that treatment of NK cells with GSK-J4 prior to co-culture with macrophages impaired the ability of NK cells to induce pro-inflammatory cytokine production in macrophages [53]. Importantly, GSK-J4 inhibition of KDM6A/B has no effect on the tumouricidal activity of NK cells, suggesting that GSK-J4 would not promote tumourigenesis as an undesirable effect [53].

Dendritic cells are innate immune cells that function as professional antigen-presenting cells, uniquely able to induce the activation and effector function of naïve T cells, forming a bridge between the innate and adaptive immune systems [54]. In addition to their antigen-presenting role, upon activation, dendritic cells can release a wide range of pro-inflammatory cytokines to promote ingress of inflammatory cells into tissues [54]. Tolerogenic dendritic cells comprise a subset of dendritic cells characterised by reduced antigen-presenting capacity and the ability to promote the generation and immunosuppressive function of regulatory T cells (Tregs) [55]. Accumulating evidence suggests that inhibition of H3K27 demethylation by GSK-J4 promotes a tolerogenic phenotype in dendritic cells to limit inflammatory and autoimmune responses. In vitro treatment of dendritic cells with GSK-J4 reduces the expression of antigen-presenting MHC class II proteins and costimulatory CD80 and CD86 molecules and increases the expression of CD103 and TGFβ1, key markers of a tolerogenic phenotype [56]. Moreover, GSK-J4 reduces the LPS-induced secretion of the pro-inflammatory cytokine IL-6 in dendritic cells [56]. Co-culture of dendritic cells and naïve CD4^+^ T cells under different polarising conditions in the presence of GSK-J4 leads to increased generation and stabilisation of Tregs with no effect on the generation of either T helper 1 (Th1) or Th2 cells [56].

When activated, naïve CD4^+^ T cells undergo differentiation into different subsets of Th cells, including Th1, Th2 and Th17 cells, each with distinctive cytokine profiles and effector functions [56]. Th17 cells constitute a subset of T helper cells, characterised by high levels of IL-17 expression, and heavily involved in mediating tissue inflammation [56]. The cytokines IL-6 and TGFβ, potentiated by other cytokines, such as IL-23, IL-21 and IL-1β, orchestrate the expansion, stability and function of Th17 cells [57]. Moreover, the transcription factor RAR-related orphan receptor γ*t*, encoded by the gene RORC, is a critical regulator of the commitment of naïve CD4 + T cells to the Th17 lineage and the expression of IL-17 [57]. Cribbs et al. (2020) [58] demonstrated an important role for KDM6A/B in regulating the in vitro differentiation, expansion and effector function of Th17 cells. They showed that GSK-J4-mediated inhibition and locked nucleic acid knockdown of each of KDM6A and KDM6B following naïve CD4^+^ T cell differentiation to Th17 cells decreased the activation of Th17, as indicated by reduced staining for CD25 and CCR4, diminished the production of IL-17 and IFNγ, and reduced the proliferative capacity of Th17 [58]. Bulk RNA-seq revealed that the inhibitory effect of GSK-J4 on differentiated Th17 cells is associated with changes in metabolic genes suggestive of Warburgian suppression of mitochondrial respiration concomitant with increased glycolysis, leading to a reduction in inflammatory cytokine production and heightened anergy [58].

### Rheumatoid arthritis

**Fig. 5:**
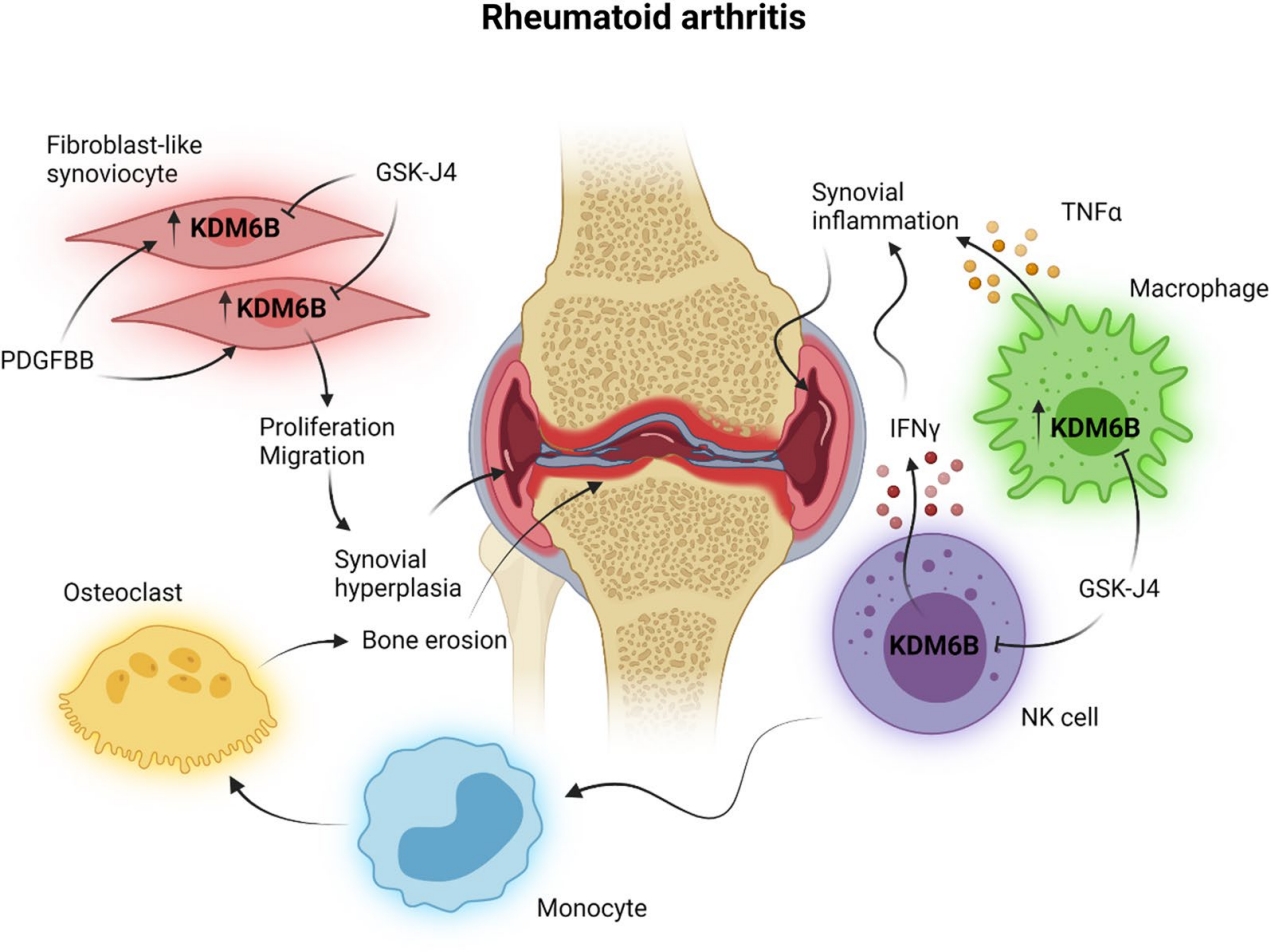
KDM6B inhibition in rheumatoid arthritis. Rheumatoid arthritis is a chronic inflammatory disease of the joints characterised by synovial hyperplasia, inflammation and bone erosion. KDM6B is implicated in platelet-derived growth factor BB (PDGFBB)-induced proliferation and migration of fibroblast-like synoviocytes, a process that underlies hyperplasia of the synovial membrane. Through inhibition of KDM6B, GSK-J4 inhibits the expansion and migratory capacity of fibroblast-like synoviocytes to attenuate synovial hyperplasia. Synovial inflammation in rheumatoid arthritis is driven by the release of pro-inflammatory cytokines, including tumour necrosis factor α (TNFα) and interferon γ (IFNγ), from macrophages and natural killer (NK) cells. GSK-J4 inhibits the production of these cytokines to dampen the inflammatory response in rheumatoid arthritis. Bone erosion in rheumatoid arthritis is mediated by bone-resorbing osteoclasts, the differentiation of which is supported by NK cells. By inhibiting KDM6B in NK cells, GSK-J4 impairs the ability of NK cells to promote the differentiation of monocytes to osteoclasts, protecting arthritic joints from bone erosion. This figure was created in BioRender.com

Rheumatoid arthritis is a chronic, inflammatory, autoimmune disease of the joints, characterised by proliferation of synoviocytes, infiltration of inflammatory cells, dominated by macrophages and CD4^+^ T cells, and destruction of both cartilage and bone [59]. Several lines of evidence suggest that H3K27 demethylases, particularly KDM6B, play a role in the pathogenesis of rheumatoid arthritis (Fig. 5). First, Jia et al. showed KDM6B upregulation in fibroblast-like synoviocytes derived from patients with rheumatoid arthritis and in the synovial tissues of rats with adjuvant-induced arthritis [60]. KDM6B upregulation was also reported in macrophages derived from patients with rheumatoid arthritis and its inhibition blocked excessive TNFα production [17]. Second, KDM6B inhibition with either GSK-J4 or siRNA-mediated knockdown inhibits fibroblast-like synoviocytes proliferation and migration in response to the mitogen platelet-derived growth factor BB (PDGFBB) [60]. PDGFBB stimulation of fibroblast-like synoviocytes upregulates KDM6B via the PI3K/Akt signalling pathway and results in its recruitment to the promoter regions of pro-proliferative genes, such as PCNA, where it demethylates H3K27me3 to enhance transcription [60]. Inhibition of KDM6B inhibits PDGFBB-induced PCNA expression by restoring trimethylation of H3K27 on PCNA promoters [60]. Finally, KDM6B inhibition with GSK-J4 attenuates the severity of arthritis in mice with collagen-induced arthritis (CIA) by reducing the serum levels of pro-inflammatory cytokines, such as IL-1β, and inflammatory cell infiltration into the joint, underpinning its potent anti-inflammatory properties [60]. Concordant with its anti-proliferative effect on fibroblast-like synoviocytes in vitro, GSK-J4 also inhibits synovial hyperplasia by suppressing the expression of PCNA in mice with CIA [60].

The synovial joints of rheumatoid arthritis patients are enriched in pro-inflammatory NK cells, which release copious amount of IFNγ in response to IL-15 stimulation and support the formation of bone-resorbing osteoclasts to promote bone erosion [47]. GSK-J4 has been shown to inhibit IFNγ production in NK cells isolated from the synovial fluids of treatment-naïve rheumatoid arthritis patients [47]. Moreover, GSK-J4 inhibited the ability of NK cells from the peripheral bloods of rheumatoid arthritis patients to promote the differentiation of monocytes into osteoclasts, which, in turn, mediate bone resorption and erosion [47]. In addition to its effects on synovial hyperplasia and inflammation in rheumatoid arthritis, GSK-J4 can also indirectly attenuate bone erosion and could be protective against joint destruction in patients.

### Osteoarthritis

**Fig. 6:**
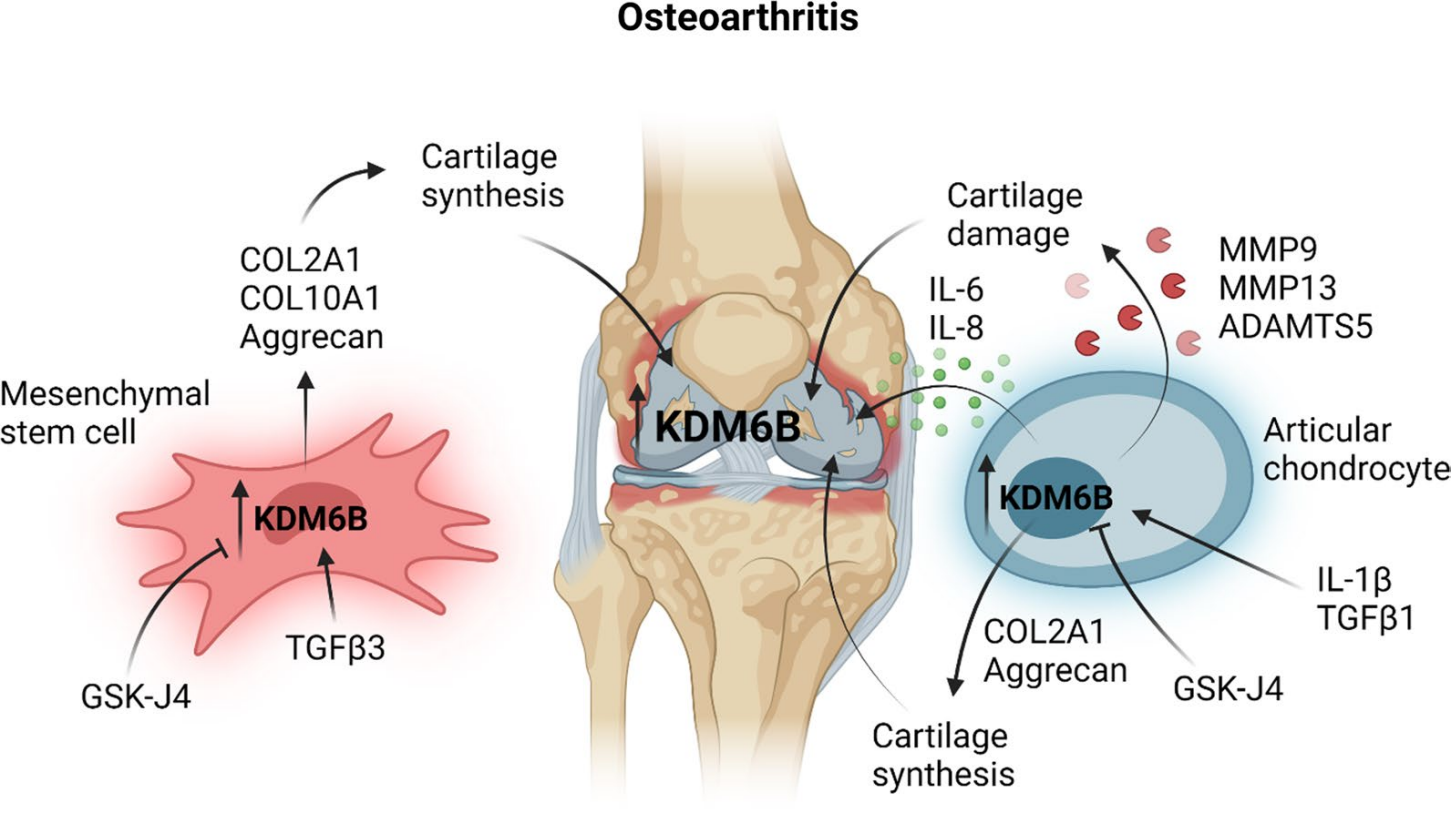
KDM6B inhibition in osteoarthritis. KDM6B is overexpressed in damaged cartilage in the knees of patients with osteoarthritis, a likely consequence of chondrocyte stimulation with IL-1β or TGFβ1. In osteoarthritis, chondrocytes upregulate the expression of the proteases MMP9, MMP13 and ADMATS5 in response to stimulation with IL-1β to promote cartilage damage and erosion. IL-1β also induces the expression of IL-6 and IL-8 in chondrocytes to create a pro-inflammatory milieu in osteoarthritis. Inhibition of KDM6B with GSK-J4 attenuates cartilage loss and inflammation in OA by suppressing these chondrocyte responses to IL-1β and TGFβ1. KDM6B also drives the expression of the cartilage components COL2A1 and aggrecan and the bone constituent COL10A1 during TGFβ3-mediated chondrogenic differentiation of mesenchymal stem cells, contributing to cartilage and bone synthesis, respectively. Inhibiting KDM6B would not promote the replacement of damaged cartilage and bone in osteoarthritis. This figure was created in BioRender.com

Osteoarthritis is the most common form of arthritis, affecting 10% of the population, and is characterised by loss of articular cartilage, a consequence of chondrocyte failure to maintain a balance between synthesis and degradation of extracellular matrix (ECM) components [61]. In addition to cartilage degradation, osteoarthritis pathogenesis involves synovial inflammation and hypertrophy and remodelling of the subchondral bone [61]. Current treatments for osteoarthritis are limited and include pain relief, physiotherapy and joint replacement surgery for patients with end-stage disease [62]. There remains a need for the development of disease-modifying therapies that can either prevent or reduce cartilage loss. Among 31 epigenetic inhibitors, the H3K27 demethylase inhibitor GSK-J4 was found to reduce the production of collagens COL2A1 and COL10A1, structural components of cartilage and bone, respectively, and the glycosaminoglycan aggrecan in primary human bone marrow-derived mesenchymal stems cells (MSCs) undergoing chondrogenic differentiation (Fig. 6) [63]. This differentiation of MSCs was accompanied by upregulation of KDM6B but not KDM6A and a concomitant reduction in H3K27 trimethylation and expression of SOX9, a key transcriptional regulator of chondrogenesis, suggesting a role for KDM6B-catalysed H3K27 demethylation in driving chondrogenesis or cartilage formation [63]. To synthesise cartilage, chondrocytes upregulate anabolic pathways to support the production of large amounts of the above-mentioned ECM proteins [64]. Chondrocyte-specific knockout of KDM6B in mice suppressed the expression of chondrocyte anabolic genes and accelerated osteoarthritis development following surgical destabilisation of the medial meniscus (DMM) [64]. Enhancing rather than inhibiting the demethylase activity of KDM6B may, therefore, normalise or augment cartilage production in osteoarthritis, replacing that which was damage or lost. Alternatively, the methyltransferase activity of EZH2 could be inhibited to offset the loss of KDM6B-mediated H3K27 demethylation in osteoarthritis. Indeed, EZH2 inhibition with the inhibitor EPZ-6438 attenuated disease progression in mice with DMM-induced osteoarthritis [65].

Strikingly, KDM6B but not KDM6A expression was found to be increased in damaged cartilage compared to undamaged cartilage derived from the same knees of osteoarthritis patients undergoing knee replacement surgery [63, 66], implicating KDM6B in articular cartilage damage and loss in osteoarthritis and contradicting the decreased KDM6B expression reported by Dai et al. (2017) [64]. Factors thought to drive KDM6B upregulation in damaged cartilage in osteoarthritis include IL-1β and TGF-β, both of which were shown to induce KDM6B expression in cultured human articular chondrocytes (HACs) [66]. Accordingly, GSK-J4 inhibited TGFβ-responsive genes, including PAI1, and diminished the IL-1β-induced expression of the pro-inflammatory cytokines IL-6 and TNFα in HACs [64, 66]. Moreover, GSK-J4 suppressed the IL-1β-mediated increase in the expression of the cartilage-degrading matrix metalloproteinases 9 (MMP9) and 13 (MMP13) and ADAMTS5 in HACs and, therefore, has the potential to attenuate cartilage loss in osteoarthritis patients [66]. Indeed, in vivo inhibition of KDM6B with GSK-J4 reversed cartilage erosion in DMM-induced murine osteoarthritis [66]. These data suggest that targeting KDM6B in osteoarthritis patients may attenuate inflammation and cartilage damage, potentially delaying disease progression, but may not promote the synthesis of cartilage to replace that which was lost since the onset of the disease.

### Inflammatory bowel disease

**Fig. 7:**
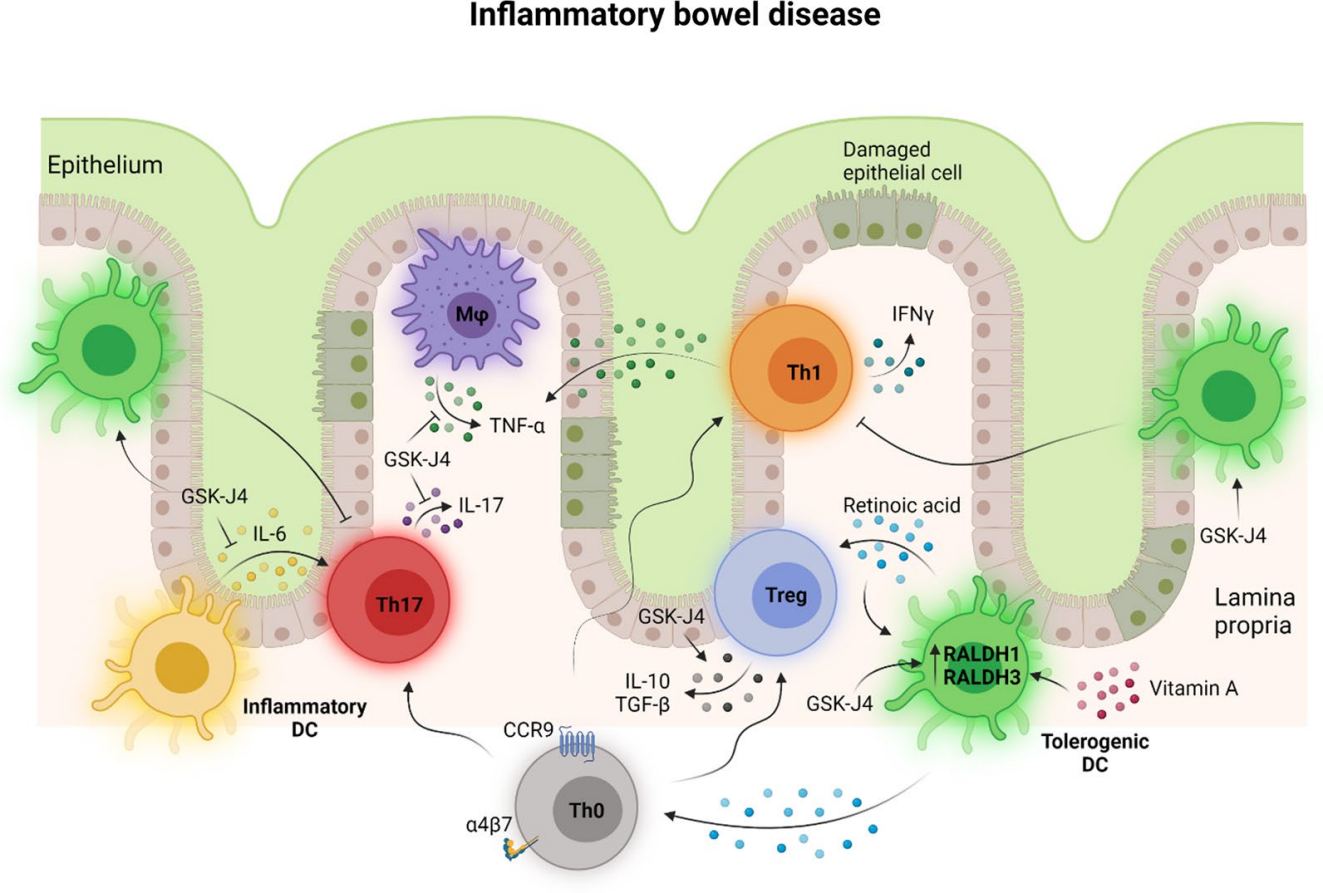
KDM6A/B inhibition in inflammatory bowel disease. Chronic gut inflammation in inflammatory bowel disease (IBD) is driven by innate and adaptive immune cells with increased reactivity to self or foreign innocuous antigens. IBD also involves the loss of regulatory T (Treg) cell-mediated immunosuppression and tolerance further disrupting immunological homeostasis in the gut. KDM6A/B inhibition with GSK-J4 in IBD restores the balance between the pro-inflammatory and anti-inflammatory arms of the adaptive immune response. GSK-J4 promotes the generation of tolerogenic dendritic cells through the upregulation of retinaldehyde dehydrogenases 1 and 3 (RALDH1 and RALDH3) and the consequent production of retinoic acid from vitamin A. Retinoic acid then promotes the recruitment of naïve CD4^+^ T (Th0) cells by inducing the expression of the gut-homing receptors CCR9 and α4β7 and their differentiation into IL-10- and TGFβ-producing Tregs with enhanced lineage stability and immunosuppressive function while restricting the generation of pro-inflammatory TNFα- and IFNγ-producing Th1 cells and IL17-producing Th17 cells. GSK-J4 also dampens gut inflammation by inhibiting the production of IL-6 by inflammatory dendritic cells and the resultant generation of Th17 cells. This figure was created in BioRender.com

Inflammatory bowel disease (IBD) refers to a complex group of chronic inflammatory disorders of the gastrointestinal tract with ulcerative colitis and Crohn’s disease being the most common [67]. Studies using murine models of inflammatory colitis and IBD patients have demonstrated that gut inflammation is predominantly mediated by Th1 and Th17 cells with increased reactivity and reduced tolerance to self or foreign antigens [68, 69]. KDM6A/B inhibition with GSK-J4 has been shown to attenuate gut inflammation in a murine model of dextran sodium sulphate (DSS)-induced colitis (Fig. 7), causing a reduction in colon shortening, bodyweight loss and the production of the inflammatory cytokines IL-6 and IL-17 in the gut mucosa [68]. Interestingly, GSK-J4 had no effect on pro-inflammatory TNFα and IFNγ or anti-inflammatory IL-10 production, implying selective inhibition of Th17-type responses in the inflamed gut [68]. Indeed, GSK-J4 reduced the frequency of inflammatory Th17 cells in the gut mucosa [68]. This effect is likely attributed to the emergence of dendritic cells with reduced inflammatory potential and increased tolerogenic activity following systemic GSK-J4 administration as intravenous injection of bone marrow-derived dendritic cells treated ex vivo with GSK-J4 similarly attenuated the severity of gut inflammation in DSS-induced colitis [68]. GSK-J4-treated dendritic cells increased the frequency of tolerance-promoting Tregs infiltrating the colonic lamina propria and the mesenteric lymph nodes while decreasing the frequency of inflammatory Th1 and Th17 cells [68]. Moreover, GSK-J4 enhanced the ability of dendritic cells to generate Tregs with higher immunosuppressive activity and lineage stability in vitro [68]. GSK-J4, therefore, has the potential to dampen the exaggerated inflammatory response in the guts of patients with IBD by restoring immunological homeostasis.

The ability of GSK-J4 to promote a tolerogenic phenotype in murine dendritic cells stems from its ability to increase de novo synthesis of retinoic acid from vitamin A, a consequence of GSK-J4-driven transcription of retinaldehyde dehydrogenase (RALDH), particularly the RALDH1 and RALDH3 isoforms [68]. GSK-J4 promoted the accumulation of the permissive epigenetic mark H3K4me3 on the promoters of RALDH1 and RALDH3 genes while reducing that of the repressive mark H3K27me3 [68]. This finding could be explained by the ability of GSK-J4, particularly at high concentrations, to inhibit the KDM5 family of lysine demethylases that target the activating H3K4me3 epigenetic mark [18]. Retinoic acid then promotes the recruitment of naïve CD4^+^ T cells to the intestinal mucosa by upregulating the gut-homing receptors CCR9 and α4β7 [68]. It also promotes the differentiation of naïve CD4^+^ T cells into Tregs while limiting the generation of Th17 cells either directly or through the induction of tolerogenic dendritic cells [68, 69].

### Atherosclerosis

Atherosclerosis is a chronic lipid-driven focal inflammatory disease of medium or large arteries [70]**.** Neointimal formation is a key event in the pathogenesis of atherosclerosis [70]. It involves the proliferation, migration and phenotypic switching of vascular smooth muscle cells (VSMCs). PDGFBB, a key growth factor that promotes VSMC proliferation and de-differentiation in atherosclerosis and other vascular remodelling diseases, significantly increases the expression KDM6B in VSMCs [71]. Moreover, KDM6B expression was shown to be upregulated in human carotid atherosclerotic plaques, implicating the H3K27 demethylase in the pathogenesis of these plaques [71]. Indeed, KDM6B inhibition with either GSK-J4 or siRNA-mediated knockdown inhibits VSMC proliferation and migration in response to PDGFBB stimulation by blocking the PDGFBB-induced increase in the expression of PCNA and cyclin D1 [71]. In vivo knockdown of KDM6B with siRNA inhibits intimal hyperplasia induced by carotid artery balloon injury in rats [71].

Macrophages play a critical role in the pathogenesis of atherosclerosis, including its initiation and progression [70]. Following monocyte infiltration into the arterial wall, differentiated macrophages scavenge modified lipids and transform into the so-called foam cells, which eventually undergo pro-inflammatory apoptosis and necrosis to destabilise the plaque [70]. Intriguingly, atherosclerotic lesions were found to be more necrotic and fibrous (rich in collagen) in high-fat diet-fed, LDLR knockout mice with bone marrow transplants from Kdm6b-null mice compared to those with bone marrow transplants from wild-type mice [72]. Bulk RNA-seq and pathway enrichment analysis revealed a significant upregulation of genes encoding cytokines chemotactic for leukocytes, including, but not limited to, CCL2. Despite this increase in the transcript expression of leukocyte chemokines, macrophage and neutrophil numbers within the atherosclerotic lesions remained unaffected [72]. These data suggest that inhibiting KDM6B may hasten plaque progression and promote rupture [72].

### Multiple sclerosis

Multiple sclerosis is an autoimmune disease characterised by the progressive loss of neurological function [73, 74]. Clinical manifestations of multiple sclerosis include paralysis, muscle spasms, optic neuritis and neuropathic pain [73]. Central to multiple sclerosis pathogenesis is the destruction of axonal myelin sheath by autoreactive CD4^+^ T cells, namely those of the pro-inflammatory Th1 and Th17 subtypes, in several regions of the brain and spinal cord [73, 74]. Opposing the actions of Th1 and Th17 cells are Tregs, and the balance between these two arms of the inflammatory response in multiple sclerosis is tipped in favour of Th1 and Th17 responses [74]. Presentation of self-antigens by dendritic cells is thought to be key to the activation of these effector CD4^+^ T cells in multiple sclerosis. Dual KDM6A/B inhibition with GSK-J4 has been shown to attenuate the severity of experimental autoimmune encephalomyelitis, an in vivo model of multiple sclerosis, in mice [56]. Treatment with GSK-J4 has been shown to promote CD4^+^ T cell polarisation towards a highly suppressive and stable Treg lineage through the induction of tolerogenic dendritic cells [56]. These data imply that GSK-J4 could potentially rebalance the pro-inflammatory and anti-inflammatory arms of the autoreactive adaptive immune response in patients with multiple sclerosis.

## H3K27 demethylase inhibition in infectious diseases

The defence employed by the host to combat infectious pathogens necessitates a highly coordinated immune response [75]. Epigenetic alterations, including histone modifications, are increasingly being recognised as important tailors of the immune response to pathogen invasion [75]. Epigenetic mechanisms are also essential for maintaining the dormancy of several viral infections [76]. Evidence has been emerging implicating the H3K27-specific demethylases KDM6A and KDM6B in the immunopathology precipitated by viral and bacterial pathogens and in the maintenance of latent viral infections. This section reviews the pre-clinical evidence in support of the use of KDM6A/B inhibition in treating various infectious diseases.

### Respiratory syncytial virus

**Fig. 8:**
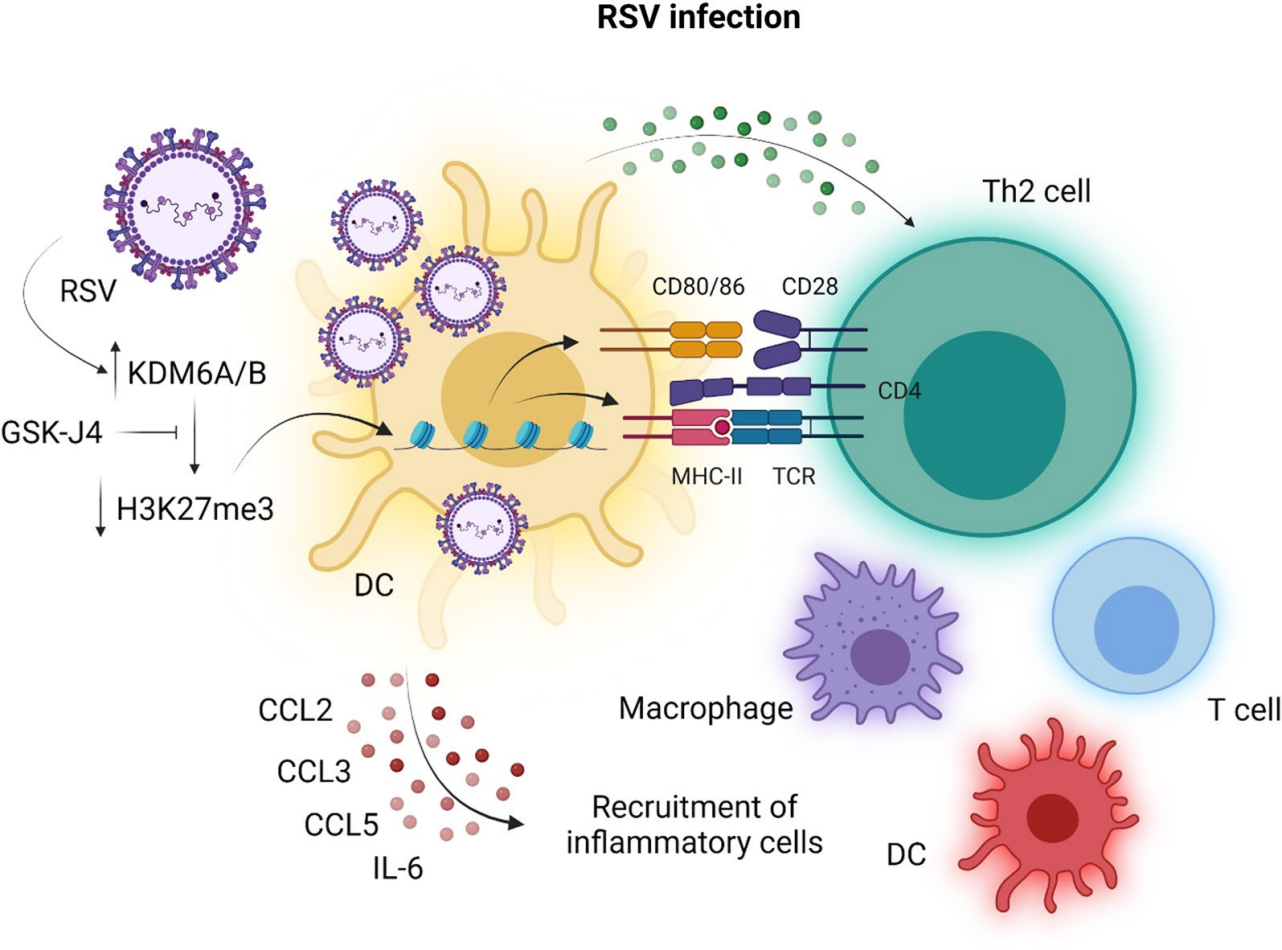
KDM6A/B inhibition in respiratory syncytial virus infection. Respiratory syncytial virus (RSV) infection is associated with the activation of dendritic cells (DCs), which promote a T helper 2 (Th2)-type response and a microenvironment within the lungs permissive for the recruitment of inflammatory cells, including macrophages, T cells and dendritic cells. DC activation by RSVs is accompanied by upregulation of the H3K27-specific demethylases KDM6A/B and a consequent decrease in H3K27 trimethylation (H3K27me3). Inhibition of KDM6A/B with GSK-J4 impairs the ability of DCs to present antigens to and activate Th2 cells through the downregulation of antigen-presenting MHC class II (MHC-II) and the costimulatory CD80/C86 molecules. GSK-J4 also inhibits the production of the pro-inflammatory cytokines and chemokines CCL2, CCL3, CCL5 and IL-6 by DCs to reduce the ingress of inflammatory cells into the lungs and attenuate the pulmonary immunopathology associated with the RSV infection. This figure was adapted from Malinczak et al. (2020) [77] and created in BioRender.com

H3K27 demethylases have been implicated in the inflammatory response to infection with respiratory syncytial virus (RSV; Fig. 8) [77]. RSV infection, the leading cause of bronchiolitis in children around the world, is associated with the activation of dendritic cells and concomitant production of pro-inflammatory cytokines that promote a T helper 2 (Th2)-type adaptive immune response [78]. Recently, Malinczak et al. (2020) demonstrated upregulation of both KDM6A and KDM6B in bone marrow-derived dendritic cells (BMDCs) as well as pulmonary dendritic cells isolated from RSV infected mice [77]. KDM6A and KDM6B upregulation was accompanied by an increase in the expression of pro-inflammatory cytokines and chemokines, including CCL2, CCL3, CCL5 and IL-6, creating a microenvironment permissive for the recruitment and activation of immune cells [77]. Dual inhibition of KDM6A and KDM6B with GSK-J4 reduced the expression of these cytokines and the antigen-presenting capacity of BMDCs as indicated by reduced expression of MHC class II proteins and the costimulatory molecules CD80/86 [77]. In vivo inhibition of KDM6A and KDM6B with GSK-J4 in mice infected with RSV reduced the numbers of pro-inflammatory CD11c^+^ dendritic cells and infiltration of activated CD4^+^ T cells within the lungs of mice infected with RSV [77]. GSK-J4 therefore has the potential to attenuate the immunopathology associated with RSV infection by dampening the exaggerated immune response to the virus.

### Human immunodeficiency virus 1

Infection with human immunodeficiency virus 1 (HIV1) is the principal cause of acquired immunodeficiency syndrome (AIDS) and affects more than 75 million people worldwide [79]. The primary targets of HIV1 are CD4^+^ T cells and, if left untreated, HIV1 can cause progressive loss of these T cells, resulting in a wide range of immunological abnormalities [79]. Despite the effectiveness of antiretroviral therapy at suppressing viral loads, upon treatment cessation, latent HIV1 within CD4^+^ T cells may rapidly reactivate [79, 80]. There are two approaches to eliminating latent HIV1 proviruses or preventing their reactivation [80]. First, the “shock and kill” approach employs cytotoxicity and/or augmented immune surveillance to eliminate the virus following pharmacological reactivation by latency reversing agents [80]. This approach, however, may fail to achieve complete reactivation of the HIV1 proviruses and induce immune-mediated viral clearance [74]. The “block and lock” strategy comprises an alternate approach that seeks to permanently inhibit the reactivation of latent HIV1 through epigenetic silencing [80].

In CD4^+^ T cells, EZH2-catalysed H3K27 trimethylation, namely at the 5’ long terminal repeat (LTR), constitutes an important silencing mechanism of HIV1 [81]. Accordingly, H3K27 demethylation by KDM6A has been demonstrated to reactivate latent HIV1 [82]. Nguyen et al. (2021) have shown that depleting KDM6A with two different shRNAs inhibits TNFα- or SAHA-induced reactivation of HIV1 in the latently HIV1-infected Jurkat T cells [82]. Chromatin immunoprecipitation (ChIP) assays revealed accumulation of RNAPII, KDM6A and H3K4me3 and depletion of H3K27me3 and EZH2 along the 5’ LTR upon HIV1 reactivation by TNFα [82]. Consistently, KDM6A knockdown caused a decrease in RNAPII occupancy throughout the HIV1 5’ LTR as well as a reduction in H3K4me3 enrichment concomitant with an increase in the local levels of H3K27me3 [82]. These data suggest that, during HIV1 reactivation, KDM6A recruitment converts the chromatin structure around the 5’ LTR to an accessible, more transcriptionally active state [82]. Finally, in Jurkat T cells, KDM6A inhibitor GSK-J4 dose-dependently attenuated TNFα- or SAHA-induced HIV1 reactivation by promoting H3K27 trimethylation, H3K4 demethylation and RNAPII binding at the 5’ LTR of the provirus [82]. GSK-J4 was also able to inhibit latent HIV1 reactivation in primary Th17 cells infected with HIV1 and CD4^+^ memory T cells from well-suppressed HIV1-infected donors [82]. Therefore, by inhibiting KDM6A, GSK-J4 has the potential to maintain viral dormancy in individuals persistently infected with HIV1.

### Herpes simplex virus 1

The neurotropic virus herpes simplex virus 1 (HSV1) establishes lifelong latency in trigeminal ganglia (TG) sensory neurones of infected individuals [83]. Occasionally, HSV-1 reactivates to produce epithelial lesions, mainly on the face but also in the eyes, causing severe keratitis. HSV-1 latency is dependent upon several repressive epigenetic marks, notably H3K27me3, that associate with the lytic genes of the latent HSV-1 genome [84]. Accordingly, evidence was provided that incriminates H3K27me3 demethylation by KDM6A/B in the reactivation of latent HSV-1 [85]. HSV-1 reactivation can be modelled in vitro by depriving latently HSV-1-infected TG neurones of nerve growth factor (NGF) [85]. Inhibition of KDM6A/B with GSK-J4 inhibits the reactivation of HSV-1 in NGF-deprived TG nerves as evidenced by reduced expression of HSV-1 lytic genes due to the accumulation of H3K27me3 on the HSV-1 genome [85]. Moreover, GSK-J4 treatment of reactivated TG nerves significantly reduces the viral yield [85]. Consequently, dual inhibition of KDM6A and KDM6B may prevent or suppress the reactivation of HSV-1 in individuals infected with the virus.

### Escherichia coli

*Escherichia coli* (*E. coli*) is a common cause of a broad spectrum of infections, ranging from non-complicated urinary tract infection to severe sepsis and septic shock [86, 87]. Currently, sepsis is associated with high mortality and is poorly managed [86]. The H3K27 demethylases KDM6A and KDM6B have been shown to play a role in the activation of the fatal inflammatory response in *E. coli*-induced sepsis [88]. This is supported by the finding that KDM6A/B inhibition with GSK-J4 promoted survival in mice with *E. coli*-induced sepsis by dampening the inflammatory response, including the production of the pro-inflammatory cytokines IL-1β, TNFα, IL-6 and CCL2, and directly suppressing the growth of *E. coli* [88]. Intriguingly, GSK-J4 was found to drive the transcription of the anti-inflammatory miR-146 by promoting the accumulation of the repressive H3K27me3 mark on its promoter region in macrophages isolated from the peritoneum of septic mice [88]. miR-146 expression is positively regulated by the p65 subunit of the key pro-inflammatory mediator NF-κB, the inhibition of which was surprisingly found to impair KDM6B binding to the promoter region of miR-146 and decrease H3K27 trimethylation [88]. This highlights a negative feedback mechanism wherein, in addition to inducing the expression of pro-inflammatory genes, NF-κB drives the expression of anti-inflammatory miR-146 to restrict exaggerated inflammatory responses [88]. Therefore, inhibiting KDM6B in septic patients may augment the anti-inflammatory arm of NF-κB signalling to calm the deleterious cytokine storm.

### Schistosomiasis

Schistosomiasis denotes a chronic disease caused by parasitic trematodes of the genus *Schistosoma* [89]. It affects, on average, more than 230 million people living in tropical and subtropical regions and results in up to 300,000 deaths annually. Schistosomiasis is largely controlled by mass treatment with the safe and potent chemotherapeutic agent praziquantel [90]. The emergence of drug-resistant schistosomal parasites, however, necessitates the search for alternative therapies. There is evidence that H3K27 demethylation by schistosomal equivalents of mammalian KDM6A and KDM6B occurs during the maturation of the parasite [91]. Although GSK-J4 has been developed as an inhibitor of mammalian KDM6A/B, inhibitory activity has been reported against schistosomal H3K27 demethylases [91]. In vitro studies revealed that GSK-J4 possesses the ability to impair motility and induce mortality in adult worms, particularly females, and schistosomula, which are particularly resistant to praziquantel [91]. Global H3K27me3 levels were unaffected following treatment of adult worms and schistosomula with GSK-J4 [91]. However, this finding does not mean that the distribution of H3K27me3 across the adult worm genome remains unaltered.

## Toxicity and tolerability of GSK-J4.

Pre-clinical evidence suggests that KDM6A/B inhibition with GSK-J4 lacks apparent toxicity, indicating that this small-molecule inhibitor may be tolerated in human patients. GSK-J4 was found to be well tolerated in mice with no weight loss or adverse, toxic effects detected in livers, kidneys, spleens, or the hematopoietic system [92, 93]. These data suggest that GSK-J4 may similarly lack hepatotoxic, nephrotoxic and bone marrow suppressive effects in human patients. GSK-J4 has also been shown to exhibit selective cytotoxicity against cancer cells with very little effects on normal cells. Sakaki et al. (2015) and Watarai et al. (2016) demonstrated that GSK-J4 concentrations up to 10 μM had no effect on the growth and viability of normal lung fibroblasts (IMR-90 cell line), whereas GSK-J4 concentrations as low as 0.5 μM were effective at inhibiting the growth and viability of ovarian cancer and non-small cell lung carcinoma cell lines [94, 95]. As reviewed by Tran et al. (2020), KDM6A plays an essential role in the differentiation of embryonic stem cells and the development of various tissues [96]. Inhibition of KDM6A with GSK-J4 may therefore be contraindicated in children and in pregnancy as it may lead to developmental abnormalities.

The ability of GSK-J4, particularly at high concentrations, to inhibit the H3K4-specific KDM5 demethylases raises the possibility that GSK-J4 may produce potential off-target effects that may be undesirable [18]. KDM5B and KDM5C catalyse the H3K4me3 epigenetic mark, which is primarily found at active promoter regions, and, in stark contrast to KDM6A/B, silence gene expression [18]. Studies often neglect the consequences of the inhibitory activity of GSK-J4 against KDM5B/C-mediated H3K4 demethylation in disease. However, evidence suggests that inhibiting KDM5B/C may be therapeutic in certain diseases, such as cancer. Indeed, KDM5B has been reported to be overexpressed in various malignancies, including those of the breast, lung, skin, liver and prostate, as reviewed by Jose et al. (2020) [97]. KDM5B preferentially binds to the promoter regions of tumour suppress genes to suppress their expression, and its inhibition with GSK-J4 may rescue the severely diminished expression of these genes in malignancies [97].

## Concluding remarks

To conclude, it is apparent that the H3K27me3-specific demethylases KDM6A and KDM6B play complex, occasionally contrasting, roles in the pathogenesis of various diseases, ranging from cancer to inflammatory, autoimmune and infectious diseases. Emerging pre-clinical evidence, both in vitro and in vivo, suggests that KDM6A/B inhibition holds immense therapeutic promise. In various types of malignancies, KDM6A predominantly serves a tumour suppressor role, whereas KDM6B functions as an oncogenic protein. Overall, however, inhibiting KDM6A/B with the small-molecule inhibitor, GSK-J4, not only suppresses the abnormal growth of cancer cells but confers upon them increased sensitivity to chemotherapeutic agents. GSK-J4 could, therefore, be used alone or better yet incorporated as an adjuvant to current chemotherapeutic regimens to impede the emergence of drug-resistant clones and ensure optimal anticancer potentials and prevention of recurrence. KDM6A and KDM6B show high homology and structural relationship, particularly in their JmjC domains. It may, therefore, be a challenge to develop a more selective inhibitor for the oncogenic KDM6B that can spare the tumour suppressor KDM6A and minimise GSK-J4’s carcinogenic potential.

Several questions remain unanswered with regards to the anticancer potentials of GSK-J4. It remains to be addressed, for example, whether KDM6A/B inhibition with GSK-J4 alters the microenvironment of solid tumours, including their stroma, vasculature and immune cell infiltration. Interestingly, Liu et al. (2020) demonstrated an essential role for KDM6B-mediated H3K27 demethylation in the hypoxic induction of the gene encoding the pro-angiogenic mediator VEGFA [98]. Moreover, KDM6B knockdown or inhibition with GSK-J4 was found to inhibit endothelial cell proliferation and tube formation under hypoxic conditions [98]. This finding suggests that, in addition to suppressing cancer cell growth, GSK-J4 may also exert anti-angiogenic effects on the tumour vasculature, starving the tumour but also impairing the delivery of chemotherapeutics. The well-documented immunomodulatory effect of GSK-J4 in inflammatory conditions suggests that KDM6A/B inhibition could alter the immune microenvironment of solid tumours in favour of an anti-inflammatory, tolerogenic milieu, possibly promoting immune evasion by tumour cells. KDM6B has also been shown to be essential for the generation of effector CD8^+^ cytotoxic T lymphocytes by enhancing chromatin accessibility in effector-associated genes (e.g. GZMB, IL2RA, ZEB2 and KLRG1) [99]. KDM6B inhibition with GSK-J4 may consequently dampen down anti-tumour cytotoxic T cell responses [99].

KDM6A/B plays an integral role in cellular responses to pro-inflammatory stimuli by controlling the expression of pro-inflammatory genes. Inhibition of KDM6A/B with GSK-J4 constitutes a potent anti-inflammatory approach and is effective against a range of conditions associated with exaggerated inflammatory responses. Moreover, in autoimmune diseases, GSK-J4 possesses the ability to dampen autoreactive immune responses and restore immunological homeostasis to inflamed tissues. Finally, KDM6A/B are key components of the epigenetic regulation of immune responses to pathogens, including viruses and bacteria, and targeted inhibition of these demethylases with GSK-J4 can attenuate the pathology associated with these responses. GSK-J4 also has the potential to maintain the dormancy of viral infections by inhibiting the KDM6A/B-mediated reactivation of the latent virus. It has yet to be addressed, however, whether GSK-J4’s immunosuppressive effects may lead to increased susceptibility to infection.

## Data Availability

Not applicable.

## Abbreviations

AML: Acute myeloid leukaemia
BCSC: Breast cancer stem cell
BMDC: Bone marrow-derived dendritic cell
CCL2: C–C motif chemokine ligand 2
CIA: Collagen-induced arthritis
CRC: Colorectal cancer
DMM: Destabilisation of medial meniscus
DSS: Dextran sodium sulphate
*E. coli*: *Escherichia coli*
EAE: Experimental autoimmune encephalomyelitis
ECM: Extracellular matrix
EZH2: Enhancer of zeste homologue 2
H3K4: Histone H3 lysine 4
H3K27: Histone H3 lysine 27
HAC: Human articular chondrocytes
HIV1: Human immunodeficiency virus 1
HSV1: Herpes simplex virus 1
IBD: Inflammatory bowel disease
IFNγ: Interferon γ
IL: Interleukin
ISC: Intestinal stem cells
JmjC: Jumonji C
JMJD3: JmjC domain-containing 3
KDM5: Lysine demethylase 5
KDM6A: Lysine demethylase 6A
KDM6B: Lysine demethylase 6B
KMT: Lysine methyltransferase
LPS: Lipopolysaccharide
LTR: Long terminal repeat
miR: MicroRNA
MMP: Matrix metalloproteinase
MS: Multiple sclerosis
MSC: Mesenchymal stem cell
NF-κB: Nuclear factor-κB
NGF: Nerve growth factor
NK: Natural killer
PC: Prostate cancer
PDGBB: Platelet-derived growth factor BB
PI3K: Phosphoinositide 3-kinase
PRC: Polycomb repressive complex
RALDH: Retinaldehyde dehydrogenase
RNA-seq: RNA sequencing
RSV: Respiratory syncytial virus
shRNA: Short hairpin RNA
siRNA: Small interfering RNA
T-ALL: T cell acute lymphoblastic leukaemia
TF: Transcription factor
TG: Trigeminal ganglia
TGFβ: Transforming growth factor β
Th: T helper cell
TNFα: Tumour necrosis factor α
Treg: Regulatory T cell
TSS: Transcriptional start site
UTX: Ubiquitously transcribed tetratricopeptide repeat on X chromosome
VSMC: Vascular smooth muscle cell
